# Measurement of spinopelvic sagittal alignment in the relaxed seated position rather than in the straight seated position is suitable for assessing spinopelvic mobility in patients before total hip arthroplasty

**DOI:** 10.1051/sicotj/2022051

**Published:** 2023-01-17

**Authors:** Yohei Ohyama, Kentaro Iwakiri, Yoichi Ohta, Yukihide Minoda, Akio Kobayashi, Hiroaki Nakamura

**Affiliations:** 1 Department of Orthopaedic Surgery, Shiraniwa Hospital Joint Arthroplasty Center 6-10-1 Shiraniwadai Ikoma City Nara 630-0136 Japan; 2 Department of Orthopaedic Surgery, Osaka Metropolitan University Graduate School of Medicine 1-4-3 Asahi-machi, Abeno-ku Osaka City Osaka 545-8585 Japan

**Keywords:** Osteoarthritis, Spinopelvic sagittal alignment, Total hip arthroplasty, Seated position, Spinopelvic mobility

## Abstract

*Purpose*: The relationship between spinopelvic mobility and dislocation in total hip arthroplasty (THA) has recently attracted attention. This study aimed to investigate the differences in sacral slope (SS) between two types of upright seated positions and to determine which seated position was appropriate for assessing spinopelvic mobility (change in SS from standing to sitting) before THA. *Materials and methods*: This prospective cohort study included 75 hips from 75 patients who had undergone primary THA. Each patient underwent preoperative lateral spinopelvic radiography in standing (st) and two seated positions: relaxed (rs) and straight (ss). The change in SS between each position (Δ) was measured. *Results*: Differences in all spinopelvic sagittal alignment parameters between the two seated positions were statistically significant (*p* < 0.001). The range, median, and mean values of ΔSS_ss-rs_ were −2.0° to 26.5°, 6.8°, and 8.3°, respectively. ΔSS_ss-rs_ was significantly correlated with SS, LLA, and PFA in the relaxed seated position (*r* = −0.52, −0.39, and 0.37; *p* < 0.001, *p* < 0.001, and *p* = 0.001, respectively), but was not correlated to these parameters in the straight seated position. Of the 52 patients with normal spinopelvic mobility in the relaxed seated position (ΔSS_st-rs_ > 10°), 24 (46%) patients were misrepresented as having a stiff spine in the straight seated position (ΔSS_st-ss_ < 10°). *Conclusion*: The change in SS from the straight to the relaxed seated position widely varied in patients before THA. The spinopelvic radiograph in the relaxed seated position is appropriate when evaluating spinopelvic mobility for preoperative planning.

## Introduction

Dislocation is a significant indicator for revision total hip arthroplasty (THA) and remains a key challenge among hip surgeons [1]. Recently, the relationship between spinopelvic mobility and dislocation due to implant impingement in THA has attracted considerable attention [2–4]. Several classifications of preoperative spinopelvic mobility and recommended cup positioning based on each classification have been reported to reduce the incidence of postoperative dislocation [3, 5, 6]. These classifications may be worth considering to help more institutions recognize patients at high risk for dislocation before performing THA. Therefore, establishing an appropriate sitting position is important for assessing preoperative spinopelvic mobility between standing and sitting postures. Our concern is that surgeons performing THA, especially those who are about to begin taking the spinopelvic sagittal alignment images as a preoperative evaluation, may not have a strictly standardized method of measuring the upright seated position. A study by the Hip-Spine Workgroup in 2019 recommended a “relaxed” or “flexed” seated position [7]. However, the methodology and terminology can be confusing, because the seated positions described in previous reports, such as relaxed or comfortable [8–12], straight or vertical [3, 4, 6, 13, 14], or flexed [15–17] positions are not standardized, and the torso posture has not been explained [2, 18]. To our knowledge, no studies have examined the differences in spinopelvic alignment between two upright seated positions before the patients underwent THAs.

We hypothesized that spinopelvic sagittal alignment significantly varies with different upright seated positions (relaxed seated vs. straight seated). This study aimed to investigate the differences in spinopelvic sagittal alignment between the two types of upright seated positions and to determine which seated position was more suitable for assessing spinopelvic mobility in patients before undergoing THA.

## Methods

This was a prospective single-center cohort study. The study protocol was approved by the Institutional Review Board, and all patients provided written informed consent before their participation. The trial was registered as a prospective cohort study in the University Hospital Medical Information Network (UMIN) [blinded].

### Study population

**Figure 1:**
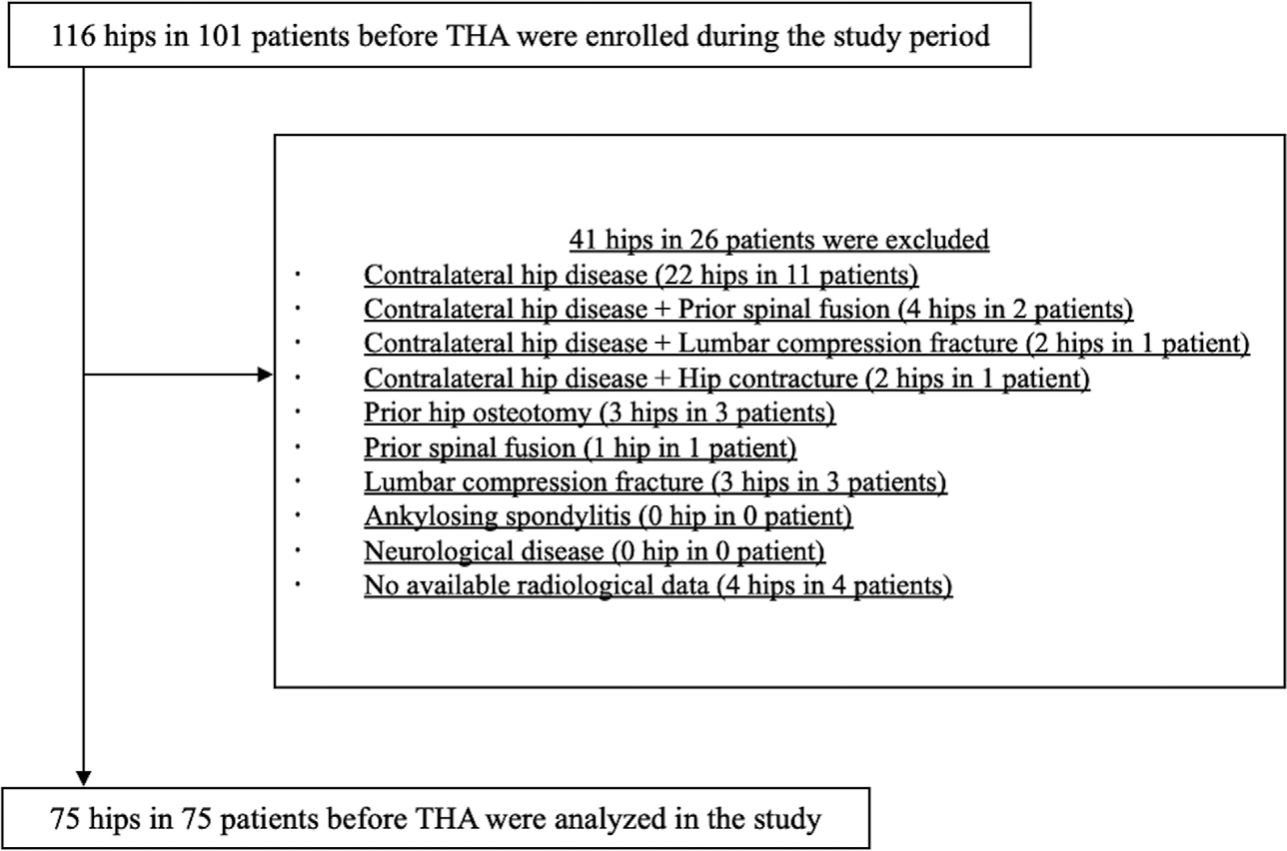
Study flow chart.

This prospective cohort study included 116 consecutive hips of 101 patients that were scheduled to undergo primary THA between April 2019 and February 2020. The exclusion criteria were as follows: (1) contralateral hip disease (e.g. osteoarthritis of the hip, osteonecrosis of the femoral head, subchondral insufficiency fractures of the femoral head, and rapidly destructive coxopathy), (2) hip disease with hip contracture affecting pelvic alignment (unable to stand or sit on a stool without aids), (3) history of hip surgery other than THA, (4) history of one or more levels spinal fusion surgery, (5) vertebral compression fracture, (6) ankylosing spondylitis, (7) neurological disease that may strongly affect spinopelvic sagittal alignment, and (8) inability to obtain complete routine preoperative radiological data. Thirty hips (15 patients) had a contralateral hip disease. Of these, four hips (two patients) had already undergone lumbar spinal fusion surgery, two hips (one patient) had lumbar compression fractures, and two hips (one patient) had contractures and developmental dysplasia (Crowe group III). Three hips (three patients) had undergone hip osteotomies on the ipsilateral hip, and one hip (one patient) had undergone lumbar spinal fusion surgery. Additionally, three hips (three patients) had lumbar compression fractures. There were no patients with ankylosing spondylitis or neurological disease. Four hips (four patients) were excluded because complete routine preoperative radiological data were not available (Figure 1). In total, 75 hips of 75 patients were included in the analysis (Figure 1). There were 66 female and nine male patients, mean age of 69.1 ± 11.1 years (range, 40–93 years) and a mean body mass index (BMI) of 23.5 ± 3.6 kg/m^2^ (range, 14.6–34.1 kg/m^2^). Of the 75 patients, 67 had osteoarthritis, five had osteonecrosis, two had subchondral insufficiency fractures of the femoral head, and one had rapidly destructive coxopathy.

### Radiographic protocol and evaluation

**Figure 2:**
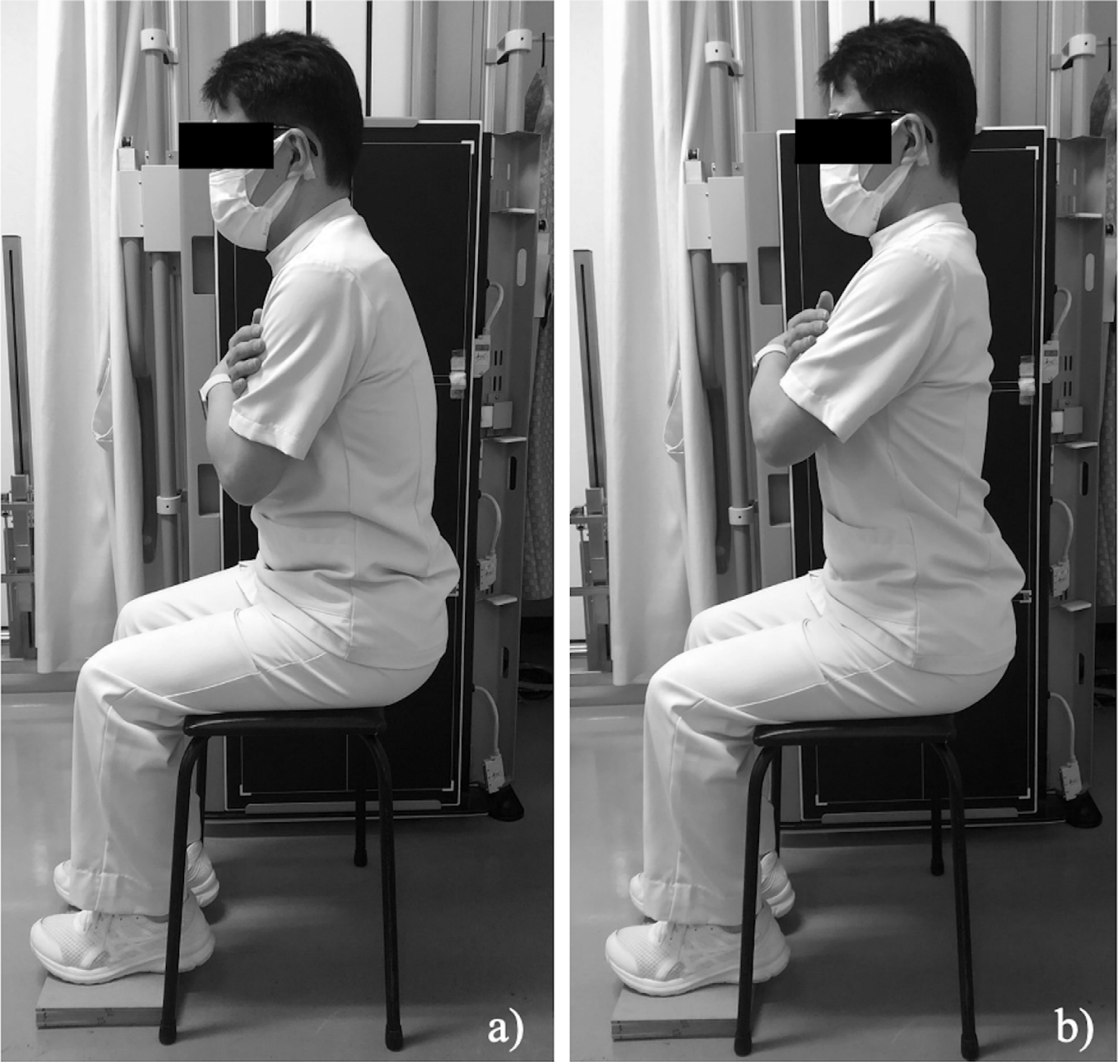
Seating positions. The patients sat back properly on the stool with their thighs parallel to the floor and their knees bent at right angles with their feet just on the boards, and follow two explicit instructions: (a) “relax and round their back” (relaxed seated position); and (b) “straighten up their back” (straight seated position).

All radiographs were made by the same criteria. For the standing lateral spinopelvic radiographs, the patients adopted a comfortable position, with their fingers resting on the clavicles as in previous studies [8]. Each patient underwent preoperative lateral spinopelvic radiography in two seated positions using a stool according to the following steps: First, the patients sat back on the stool with their thighs approximately parallel to the floor and their knees bent at approximately right angles, and five-millimeter-thick boards were placed on the floor to adjust the height so that the soles of the feet just touched the ground. The patients were instructed to follow two explicit instructions: (1) to relax and round their back (relaxed seated position) (Figure 2a); and (2) to straighten their back (straight seated position) (Figure 2b). The examiner then instructed the patient to sit with the head and the buttocks aligned with the line of gravity so that they would not lean forward and backward. All lateral spinopelvic radiographs captured the pelvis, including the spine and proximal femur, on three vertically lined cassettes placed adjacent to the patient’s right side (Figure 2). The X-ray beam was centered in the middle of the cassettes and irradiated to obtain maximum overlap of the left and right anterior superior iliac spines; the radiographic source-film distance was 250 cm. The sacral slope (SS), pelvic tilt (PT), pelvic incidence (PI), lumbar lordotic angle (LLA), and pelvic-femoral angle (PFA) were measured as spinopelvic sagittal alignment parameters. The changes in each parameter between each position (Δ) were noted:

$$\Delta X_{\mathrm{st}-\mathrm{rs}}\;=\;X_{\mathrm{standing}}–X_{\mathrm{relaxed}\;\mathrm{seated}}$$

$$\Delta X_{\mathrm{st}-\mathrm{ss}}=\;X_{\mathrm{standing}}–X_{\mathrm{straight}\;\mathrm{seated}}$$

$$\Delta X_{\mathrm{ss}-\mathrm{rs}}=X_{\mathrm{straight}\;\mathrm{seated}}–X_{\mathrm{relaxed}\;\mathrm{seated}}$$

with X being SS, PT, LLA, and PFA [19]. SS was the angle between the superior endplate of S1 and a horizontal line, PT was the angle between a line drawn from the center of the superior endplate of S1 to that of the femoral head and a vertical line, PI was calculated as the sum of SS and PT in the standing position, LLA was the angle between the superior endplates of L1 and S1, and PFA was the angle between a line drawn from the center of the superior endplate of S1 to that of the femoral head and a line drawn parallel to the proximal femoral diaphysis. We focused on SS as the primary dependent variable to assess spinopelvic mobility, because previous studies reported that SS and ΔSS between standing and sitting are the most optimal, practical, and easily measured indicators of dislocation risk [3–5, 7]. Patients were classified into four groups according to the Hip-Spine Classification system to assess spinal alignment (PI-LLA) and spinopelvic mobility (ΔSS) as follows:

Group 1A is a patient with normal spinal alignment (PI-LLA in the standing position < 10°) and normal spinopelvic mobility (ΔSS_st-rs_ > 10° or ΔSS_st-ss_ > 10°), group 1B is a patient with normal spinal alignment (PI-LLA in the standing position < 10°) and a stiff spine (ΔSS_st-rs_ < 10° or ΔSS_st-ss_ < 10°), group 2A is a patient with flatback deformity (PI-LLA in the standing position > 10°) and normal spinopelvic mobility (ΔSS_st-rs_ > 10° or ΔSS_st-ss_ > 10°), group 2B is flatback deformity (PI-LLA in the standing position > 10°) and a stiff spine (ΔSS_st-rs_ < 10° or ΔSS_st-ss_ < 10°) [5].

We measured all radiographic data using computerized picture archiving and communication systems technology (RapideyeCore™; Canon Medical Systems Corporation, Tochigi, Japan). The interobserver and intraobserver variabilities in parameter measurements (SS, LLA, and PFA in the relaxed seated position) were assessed using the interclass correlation coefficient with a random number table of 12 selected subjects (10% of the total number of subjects) and two blinded observers. We repeated the measurements every two months.

### Statistical analysis

All data were collected and analyzed using BellCurve for Excel ver. 3.20 (Social Survey Research Information Co., Ltd., Tokyo, Japan). Differences between the two types of spinopelvic sagittal alignment were statistically analyzed using the paired *t*-test or Wilcoxon signed-rank test. Single regression analysis and multiple regression analysis with a forced data entry method were conducted to determine factors predicting ΔSS. Correlations between ΔSS and other data were analyzed using Spearman’s rank correlation coefficient (*r*). The coefficient values were characterized as follows: 0.00–0.19, poor, if any; 0.20–0.39, fair; 0.40–0.59, moderate; 0.60–0.79, good; and 0.80–1.00, high/strong [8]. A *p* < 0.05 was considered statistically significant.

A power analysis using computer software (G* Power 3.1.9.2; Heinrich-Heine-Universität Düsseldorf, Düsseldorf, Germany) was performed to determine sample size to detect a significant difference of 5°, with a standard deviation (SD) of the change in sacral slope between the two seated positions of 13.6° based on a previous study [8]. A sample size of 61 hips or more was determined to provide a power of 80%, with the two-sided alpha set at 0.05 using a dependent *t*-test.

## Results

### Spinopelvic sagittal alignment in the two seated positions

There was an excellent agreement between intraobserver and interobserver reliabilities for SS, LLA, and PFA (0.993 vs. 0.962, 0.995 vs. 0.956, and 0.989 vs. 0.911, respectively). The mean values and range of SS, PT, PI, LLA, and PFA in each position are shown in Table 1. The differences in all spinopelvic sagittal alignment parameters except PI between the two seated positions were statistically significant (*p* < 0.001) (Table 1).

**Table 1:** Spinopelvic sagittal alignment parameters in each position and comparison of each parameter between the relaxed and straight seated positions.

	Standing position	Relaxed seated position	Straight seated position	*p*-value †
Sacral slope (°)	35.5 ± 10.3 (−8.0 to 57.8)	18.1 ± 12.9 (−29.2 to 44)	26.5 ± 11.9 (−26.7 to 49.5)	<0.001*
Pelvic tilt (°)	20.8 ± 9.3 (−8.0 to 57.8)	39.0 ± 12.9 (16.1–74.5)	30.7 ± 11.6 (8.8–68.9)	<0.001*
Pelvic incidence (°)	56.3 ± 11.0 (−32.1 to 81.8)	N/A	N/A	N/A
Lumbar lordotic angle (°)	39.4 ± 17.5 (−37.7 to 73.7)	19.5 ± 16.3 (−35 to 55.5)	29.5 ± 15.9 (−37.5 to 62.2)	<0.001**
Pelvic-femoral angle (°)	189.9 ± 12.1 (161.8–216.8)	141.0 ± 15.8 (110.1–190.4)	132.7 ± 15.1 (99.8–189.6)	<0.001**

All values are represented as mean ± standard deviation (range).

† Comparison of spinopelvic sagittal alignment parameters between the relaxed and straight seated positions.

* Wilcoxon signed-rank test.

** Paired *t*-test.

N/A, not applicable.

### Change in sacral slope from the straight to the relaxed seated position (ΔSS_ss-rs_)

**Figure 3:**
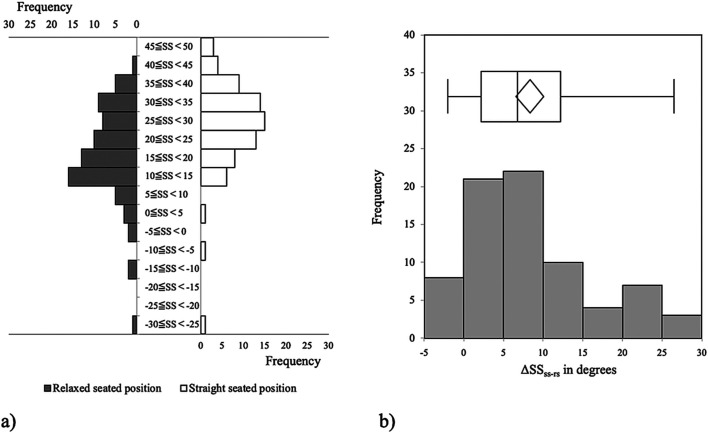
The distributions of sacral slope (SS) in each seated position (a) and the change in SS from the straight to the relaxed seated position (ΔSS) (b) are shown. ΔSS is not normally distributed. The range, median, and mean values are −2.0° to 26.5°, 6.8°, and 8.3°, respectively.

Figure 3 showed the distributions of SS in each seated position and ΔSS_ss-rs_. ΔSS_ss-rs_ was widely distributed, with range, median, and mean values ranging from −2.0° to 26.5°, 6.8°, and 8.3°, respectively (Figure 3b).

**Figure 4:**
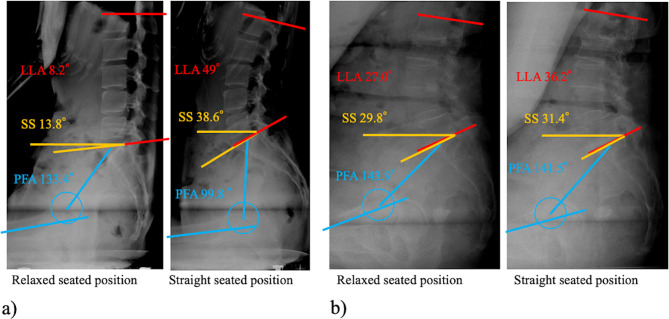
Typical radiographs showing quite the opposite change in spinopelvic sagittal alignments. (a) A 46-year-old female with a large change in sacral slope. Features: posterior rotation of the pelvis and lumbar kyphosis in the relaxed seated position. (b) A 78-year-old female patient with a small change in sacral slope. Features: anterior rotation of the pelvis and lumbar lordosis in the relaxed seated position.

There was a good correlation between ΔSS_ss-rs_ and ΔLLA_ss-rs_ (*r* = 0.79, *p* < 0.001). Similarly, there was a good correlation between ΔSS_ss-rs_ and ΔPFA_ss-rs_ (*r* = –0.76, *p* < 0.001). The correlation coefficients between ΔSS_ss-rs_ and spinopelvic sagittal alignment (SS, LLA, and PFA) in the relaxed seated position were fair (*r* = −0.52, −0.39, and 0.37; *p* < 0.001, *p* < 0.001, *p* = 0.001, respectively) (Table 2, Figure 4). The correlations between ΔSS_ss-rs_ and spinopelvic sagittal alignment (SS, PI, LLA, and PFA) in the standing and straight seated position were not significant (Table 2). A multiple regression analysis adjusted for sex, age, BMI, and spinopelvic sagittal alignment parameters other than SS (LLA, and PFA) in the relaxed seated position revealed that LLA and PFA in the relaxed seated position were significant predictors for ΔSS_ss-rs_ (*β* = −0.25 and 0.27; *p* = 0.04 and *p* = 0.04; Adjusted *R*^2^ = 0.16). There were no significant predictors in a multiple regression analysis adjusted for sex, age, BMI, and spinopelvic sagittal alignment parameters other than SS (LLA, and PFA) in the straight seated position.

**Table 2:** Correlations with the change in sacral slope from the straight to the relaxed seated position (ΔSS_ss-rs_).

		Correlation coefficient (*r*)*	*p*-value*
Age		−0.19	0.09
BMI		−0.15	0.19
Standing position			
	SS	0.09	0.45
	PI	0.03	0.78
	LLA	−0.01	0.94
	PFA	−0.02	0.86
Relaxed seated position			
	SS	−0.52	<0.001
	LLA	−0.39	<0.001
	PFA	0.37	0.001
Straight seated position			
	SS	0.13	0.26
	LLA	0.03	0.79
	PFA	0.02	0.85
ΔLLA_ss-rs_		0.79	<0.001
ΔPFA_ss-rs_		−0.76	<0.001

* Spearman’s rank correlation.

ΔSS_ss-rs_ was significantly correlated with the spinopelvic sagittal alignment parameters (i.e., SS, LLA, and PFA) in the relaxed seated position, ΔLLA_ss-rs_, and ΔPFA_ss-rs_.

SS, sacral slope; PI, pelvic incidence; LLA, lumbar lordotic angle; PFA, pelvic-femoral angle; BMI, body mass index; ΔSS_ss-rs_, change in sacral slope from the straight to the relaxed seated position; ΔLLA_ss-rs_, change in lumbar lordotic angle from the straight to the relaxed seated position; ΔPFA_ss-rs_, change in pelvic-femoral angle from the straight to the relaxed seated position.

### Spinal alignment and spinopelvic mobility from the standing to two types of seated positions

**Figure 5:**
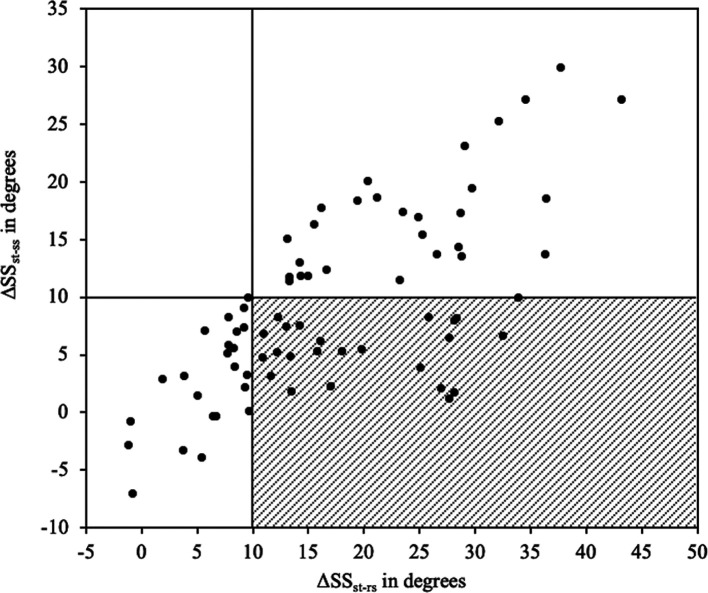
Scatterplot of spinopelvic mobility (ΔSS) from the standing to the relaxed or straight seated position. Fifty-two patients with normal spinopelvic mobility in the relaxed seated position (ΔSS_st-rs_ > 10°) are in the right quadrant. Twenty-four (46%) of them are classified as having a stiff spine (ΔSS_st-ss_ < 10°) in the straight seated position (shaded area in the lower right quadrant), but they would not actually have stiff spines. ΔSS_st-rs_, change in sacral slope from standing to relaxed seated; ΔSS_st-ss_, change in sacral slope from standing to straight seated.

We categorized into four groups according to the Hip-Spine Classification system (Table 3) [5]. The percentage of patients with flatback deformity (group 2A + 2B) was 65%, a higher percentage than in a previous report [5], presumably due to the higher average age of the patients in this study. The percentage of patients with a stiff spine (group 1B + 2B) was 30% in the relaxed seated position, and 61% in the straight seated position, which indicated an abnormal distribution. None of the 29 patients with normal spinopelvic mobility in the straight seated position (ΔSS_st-ss_ > 10°) was classified as having a stiff spine in the relaxed seated position (Table 3, Figure 5). Fifty-two patients were classified as having normal spinopelvic mobility in the relaxed seated position (ΔSS_st-rs_ > 10°); however 24 (46%) of them were classified as having a stiff spine (ΔSS_st-ss_ < 10°) in the straight seated position and possibly not actually having a stiff spine (Table 3, Figure 5).

**Table 3:** Number and percentage of patients in each of the four groups according to the Hip-Spine Classification.

	Group	1A	1B	2A	2B
Relaxed seated position	Classification	PI-LLA < 10° and ΔSS_st-rs_ > 10°	PI-LLA < 10° and ΔSS_st-rs_ < 10°	PI-LLA > 10° and ΔSS_st-rs_ > 10°	PI-LLA > 10° and ΔSS_st-rs_ < 10°
	Patients, *n* (%)	19 (25)	7 (9)	33 (44)	16 (21)
Straight seated position	Classification	PI-LLA < 10° and ΔSS_st-ss_ > 10°	PI-LLA < 10° and ΔSS_st-ss_ < 10°)	PI-LLA > 10° and ΔSS_st-ss_ > 10°	PI-LLA > 10° and ΔSS_st-ss_ < 10°
	Patients, *n* (%)	11 (15)	15 (20)	18 (24)	31 (41)
Vigdorchik et al. [5]	Patients, *n* (%)	987 (47)	232 (11)	715 (34)	147 (7)

PI-LLA, pelvic incidence minus lumber lordosis angle; ΔSS_st-rs_, change in sacral slope from the standing to the relaxed seated position; ΔSS_st-ss_, change in sacral slope from the standing to the straight seated position.

## Discussion

The most important finding of this study was that spinopelvic sagittal alignment varied significantly with different sitting positions, and the change in sacral slope (ΔSS_ss-rs_) widely varied between the two types of upright seated positions (i.e., −2.0° to 26.5°) before THA. Several recent studies have focused on the relationship between spinopelvic mobility and different seated positions: relaxed and flexed [19–21]. This is the first study to compare and validate spinopelvic sagittal alignment between two upright seated positions before the patients underwent THAs. The mean SS in the upright seated positions varied among studies; the minimum and maximum values were 11.7° and 25.7°, respectively [12, 21]. Behery et al. advocated that flexed sitting imaging may emphasize the spinopelvic mobility of patients with hip osteoarthritis more than relaxed imaging [21]. However, the mean SS in the relaxed seated position in their study was 25.7°, similar to that obtained in this study for the straight seated position and approximately 10° greater than that in the relaxed seated position in previous studies [8, 9, 20]. The difference would be clinically meaningful and may be attributed to the non-standard seating methods or unclear instructions regarding sitting during radiographic imaging. Different patients may have different postures in which they feel relaxed.

Although there are multiple ways to categorize the spinopelvic relationship and define an at-risk condition for THA patients, it would be beneficial for surgeons to develop a classification system that identifies patients at high risk for postoperative dislocation and allows for appropriate preoperative evaluations and planning [3, 5–7, 22, 23]. A prospective multicenter study showed that THA performed via the posterior approach, with patient-specific component positioning determined according to a novel Hip-Spine Classification system using PI-LL and change in SS, resulted in low dislocation rates, even in high-risk patients [5]. In this study, the change in SS between the functional seated positions (ΔSS_ss-rs_) increased with greater posterior rotation of the pelvis (smaller SS), lumber kyphosis (smaller LLA), and larger PFA in the relaxed seated position, regardless of PI, the position-independent parameter of the sagittal morphology of pelvis (Figure 4). The spinopelvic sagittal alignment parameters in the relaxed seated position moderately predict those in the straight seated position. Moreover, this study found that approximately half (46%) of patients were classified as having a stiff spine when assessing spinopelvic mobility using the spinopelvic sagittal alignment parameters in the straight seated position. Conversely, none were misclassified in the same patient cohort when assessed in the relaxed seated position. Therefore, when using the change in SS as a preoperative planning tool, the spinopelvic lateral radiograph in the straight seated position may misrepresent spinopelvic mobility, making the patient’s spine appear stiffer. We believe that measuring spinopelvic sagittal alignment in the relaxed seated position may be appropriate for assessing spinopelvic mobility in patients before THA. Understanding the tendency of spinopelvic sagittal alignment, especially in patients with large ΔSS_ss-rs_ can help medical personnel take more care in instructing patients how to sit for the assessment of spinopelvic mobility.

This study has a few key limitations. First, we investigated only spinopelvic sagittal alignment before THA, not its change after THA. Previous studies have reported that spinopelvic mobility is minimally changed by THA [9–11]. Yun et al. showed that although preoperative SS correlated strongly with postoperative SS in the supine and standing positions and change in SS was minimal by THA overall, there was high variability to be clinically relevant, especially in the sitting position and spinopelvic mobility, over ±7° changes [9]. Future studies are needed to evaluate whether THA with consideration of the spinopelvic sagittal alignment makes a difference in pre-and postoperative spinopelvic mobility and patient outcomes. However, there have been several studies to predict impingement risk or identify high-risk patients with preoperative THA spinal-pelvic alignment parameters [5, 6, 23]. Tezuka et al. [6] recently reported that preoperative PFA was the best predictor of non-conformance to the functional safe zone defined by the combined sagittal index (the sum of cup anteinclination and PFA) postoperatively. In addition, as mentioned earlier, several preoperative spinopelvic sagittal alignment classifications have already been presented to reduce postoperative impingement. Therefore, first and foremost, standardization of preoperative spinopelvic radiography is essential for comparison with other studies and to improve the reproducibility of evaluations. Another major concern is the change in spinopelvic sagittal alignment over a long time. Tamura et al. [24] describe a posterior pelvis rotation in the standing position over ten years following primary THA. Therefore, establishing the cup orientation is critical to minimize dislocation by considering the relationship between preoperative and long-term postoperative spinopelvic mobility. Finally, selection bias may have been introduced because of the observational nature of this radiography-based study. However, radiographic data were prospectively collected according to a strict protocol based on the hypothesis that spinopelvic sagittal alignment varies significantly with different upright seated positions. Moreover, we excluded patients with a history of surgery or disease that could strongly affect spinopelvic sagittal alignment. The demographic data of patients in this study were similar to those in a previous report on THA in Japanese individuals [8].

In conclusion, the change in sacral slope between two types of upright seated positions (ΔSS) exhibited a wide range of values (−2.0° to 26.5°) in patients before THA and could be predicted using the spinopelvic sagittal alignment parameters in the relaxed seated position. The relaxed seated position more adequately classified the spinopelvic mobility compared to the straight seated position when obtaining preoperative spinopelvic radiographs. Therefore, we recommend standardizing the terminology and methodology and providing explicit instructions on the relaxed seated position to patients during radiographic imaging.
